# Confabulations in Cases of Dementia: Atypical Early Sign of Alzheimer’s Disease or Misleading Feature in Dementia Diagnosis?

**DOI:** 10.3389/fpsyg.2020.553886

**Published:** 2020-09-29

**Authors:** Elisabetta Belli, Valentina Nicoletti, Claudia Radicchi, Joyce Bonaccorsi, Simona Cintoli, Roberto Ceravolo, Gloria Tognoni

**Affiliations:** Neurology Unit, Department of Clinical and Experimental Medicine, University of Pisa, Pisa, Italy

**Keywords:** spontaneous confabulations, provoked confabulation, Alzheimer’s disease, frontotemporal dementia, self-awareness, anosognosia, dementia, confabulations

## Abstract

Confabulations, also known as false memories, have been associated with various diseases involving mainly the frontal areas, such as Wernicke–Korsakoff syndrome or frontal epilepsy. The neuropsychological dysfunctions underlying mechanisms of confabulation are not well known. We describe two patients with memory impairment and confabulations at the onset speculating about neuropsychological correlates of confabulations and self-awareness. Both patients, a 77-year-old woman and a 57-years-old man, exhibited confabulations as first symptom of cognitive decline. She later developed memory impairment without awareness of her memory deficits and her cognitive and imaging profile suggested an amnesic mild cognitive impairment due to Alzheimer’s disease (AD). Unlike her, he developed a prevalent involvement of frontal functions despite a clear consciousness of his cognitive deficits. However, the clinical diagnostic hypothesis of behavioral variant of frontotemporal dementia was not supported by imaging findings, which suggested AD. Both patients underwent neuropsychological evaluation including the Confabulation Battery. Despite that the exact anatomical correlation of confabulations is still not defined, imaging data shown by our patients is consistent with recent theories according to which at the origin of confabulatory tendency in AD there is an impairment of the connections between crucial hubs in frontal and mediotemporal areas, mainly involving the right hemisphere. Besides, it would be reasonable to hypothesize that self-awareness and confabulations should not be considered as necessarily associated dimensions.

## Introduction

Confabulations are defined as actions and verbal statements unintentionally incongruous to the patient’s history, background, and present and future situation (Dalla Barba and Decaix, 2009). Confabulations are commonly distinguished in provoked, if produced in response to direct questions, and spontaneous, if independent from any external stimulus (Kopelman, 2010). Spontaneous confabulations have been linked to frontal lobe pathology such as in Wernicke–Korsakoff syndrome, subarachnoid hemorrhage due to the rupture of anterior communicating artery aneurysms, and frontal lobe epilepsy (Fujikawa et al., 2016).

Many hypotheses have been formulated over time. According to the “temporality theory,” confabulations, particularly spontaneous ones, are true memories displaced in time, resulting from the mind failure to recognize the correct temporal order of memories. On the other hand, according to the “strategic retrieval hypothesis” confabulations, particularly provoked ones (Schnider et al., 1996) result from the attempt to recollect information from a deficient memory (Gilboa et al., 2006).

While spontaneous confabulation collection is essentially anamnestic, provoked confabulations can be measured through the Confabulation Battery (CB) (Dalla Barba et al., 2019).

Confabulations are rarer in Alzheimer’s disease (AD), especially if compared with other degenerative dementias such as frontotemporal dementia (FTD) (Abbate et al., 2016), and have been related to the degree of cognitive impairment assessed by Mini Mental State Examination (MMSE). Moreover, provoked confabulations are more frequent in mild stages of the disease, while spontaneous confabulations are typically observed in advanced stages (El Haj and Laroi, 2017).

We describe the cases of two patients with an unusual neuropsychiatric presentation at disease onset speculating about the possible anatomical and neuropsychological correlates of confabulations and self-awareness.

## Materials and Methods

Both patients were visited at the memory outpatient clinic of Pisa Hospital. Cognitive performances and neuropsychiatric disturbances were assessed respectively through MMSE (Measso et al., 1993) (cutoff > 23/30) and Neuropsychiatric Inventory test (NPI) (Cummings, 1997). Self-consciousness was assessed through the Anosognosia Questionnaire for Dementia (AQ-D) (Gambina et al., 2015).

Patients went through neuropsychological assessment both at first visit (T1) and respectively after 2 and 3 years (T3). At T3, the CB was also administered to detect provoked confabulations, while spontaneous confabulations were both anamnestically reported by relatives and produced during medical visits.

Patients also underwent neuroimaging scans, comprehensive of a morphological exam [magnetic resonance imaging (MRI) – or computerized tomography (CT)] and a functional one [cerebral positron emission tomography with fluorodeoxyglucose (FDG-PET)].

**FIGURE 1:**
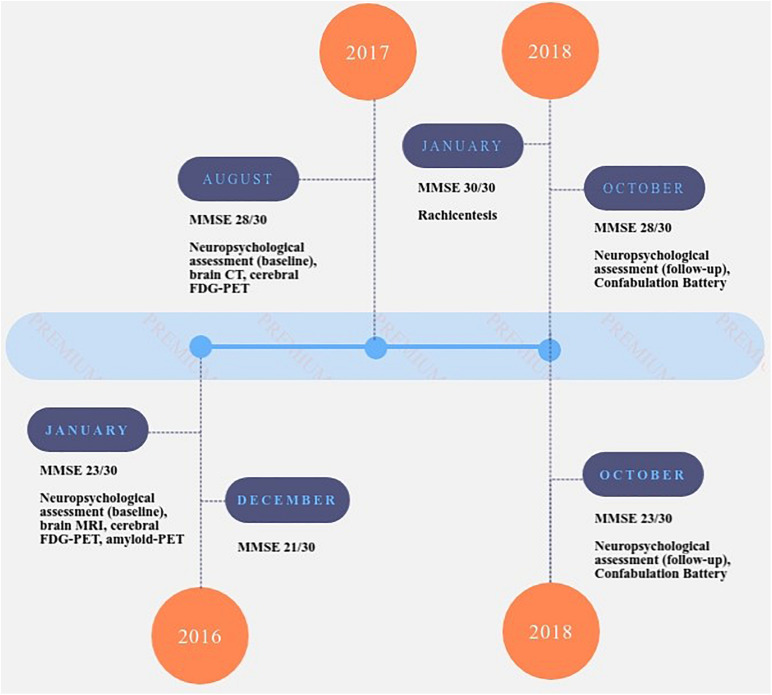
Timeline of events for patient A.R. (upper part of the figure) and A.G. (lower part of the figure). MMSE: Mini Mental State Examination (Folstein et al., 1975; Measso et al., 1993); NPI: Neuropsychiatric Inventory test (sub-items – sub-item delusions, hallucinations, agitation, depression, anxiety, euphoria, apathy, disinhibition, irritability, aberrant motor, night-time behavior, appetite) (Cummings, 1997).

The first patient underwent lumbar puncture for assessment of biomarkers (Aβ42, t-tau, and p-Tau181), which were measured with commercially available enzyme-linked immunosorbent assays (INNOTEST (IT) ELISAs Fujirebio Europe, Ghent, Belgium). The second patient performed amyloid-PET. In Figure 1, the timeline of events for each patient is reported.

Approval was obtained from the local ethical standards committee on human experimentation, and written informed consent was obtained from subjects before enrolment. The study has been performed in accordance with the ethical standards laid down in the 1964 Declaration of Helsinki and its later amendments.

## Case Description

### Case 1

A.R.’s first visit was in August 2017 (T1), at the age of 77. She was a housewife with 13 years of education, with no relevant previous pathologies. Her husband reported memory impairment for 1 year, spatial disorientation, and spontaneous confabulations, which emerged in particular while seeing photos/images of cities around the world. She started to narrate journeys she never made. Her husband also reported some behavioral changes (apathy and irritability with lack of awareness of the disease). When asked about her alleged journeys by doctors, she gave plausible explanations about discrepancies, giving reasons also for the lack of proofs (such as photos). Noteworthy is her tendency to justify her confabulations when facing the inconsistency of her stories: these additional justifications take the name of secondary claims and can be considered confabulations themselves (Turner and Coltheart, 2010).

Neurological examination was normal, MMSE was 27.86/30 (corrected score), and NPI was 12/144 (irritability and apathy predominant sub-items); during the interview, false memories and confabulations were evident. Neuropsychological assessment was mostly normal (Table 1, assessment at baseline). Brain CT showed a subtle enlargement of temporal horns due to adjacent parenchymal atrophy; cerebral FDG-PET highlighted a mild hypometabolism in the temporo-mesial and posterior dorsolateral parietal regions, in the precuneus bilaterally, and in the temporo-lateral region on the right side.

**TABLE 1:** Results of neuropsychological evaluation performed at baseline and during a follow-up visit.

Neuropsychological domain	Cutoff	A.R.		A.G.	
		Baseline	Follow-up	Baseline	Follow-up
**Anterograde memory**					
Digit span	≥3,75	4,75	5,75	5,75	5,75
Corsi span	≥3,50	3,75	4,25	4,75	3,75
RAVLT: immediate recall	>28,53	43,2	37,2	23*	14,6*
RAVLT: delayed recall	>4,69	6,9	3,9*	1,5*	0*
ROCF: immediate recall	>6,44	7,3	7,4	4*	0,5*
ROCF: delayed recall	>6,33	5,9*	0*	7,1	0*
Prose memory: immediate recall	>3,10	4,8	7,2	3,3	0*
Prose memory: delayed recall	>2,39	4,5	0*	0*	0*
**Attention**					
Visual search	>31	51,75	56,75	37,5	32
WAIS: digit symbol substitution test	>5	14		9	2*
**Stroop interference test**					
Time	≤36,91	6,5	4,5	50,75*	108,5*
Errors	≤4,23	0	−0,75	0	3,75
**Trail-making test**					
Test A	≤127		52	61	39
Test B	≤294		61	119	315*
B-A	≤163		9	57	276*
**Executive function**					
Raven’s matrices	>18,96	29,8		22,3	14,8*
Word fluency (category)	>24			31	19*
Word fluency (letter)	>17,35	45,5	32,5	36,8	24,3
Frontal Assessment Battery	≥13,5	16,2	15,2		9,5*
CLOX 1	≥10	12	12	8*	11
**Constructional praxis**					
ROCF copy	>23,76	31,2	34,2	31,3	27,2
CLOX 2	≥12	14	14	14	12
**Anosognosia**					
AQ-D score		P: 8 Cg:20	P: 7 Cg:20	P: 5 Cg: 7	P: 5 Cg: 7

Short-term memory was evaluated with digit span forward (Orsini et al., 1987) and corsi test forward (Orsini et al., 1987). Retrospective memory was assessed using the Rey Auditory Verbal Learning Task (RAVLT) (Carlesimo et al., 1996) and the Rey–Osterrieth Complex Figure Test (ROCF) (Carlesimo et al., 2002), immediate and delayed recall. Executive functions and Attention were assessed using the Frontal Assessment Battery (FAB) (Apollonio et al., 2004), the phonemic verbal fluency (Carlesimo et al., 1996), Trail Making Test (TMT) (Giovagnoli et al., 1996), WAIS (Orsini and Laicardi, 1997), and the Stroop Interference Test (Caffarra et al., 2002). Constructional praxis was assessed with the copy of ROCF (Carlesimo et al., 2002). Visuospatial abilities and non-verbal intelligence were measured with the Raven Colored Progressive Matrices (CPM-47) (Carlesimo et al., 1996). Corrected scores for age and education are reported. The * symbol indicates that the score is compromised. Anosognosia was assessed through the Anosognosia Questionnaire for Dementia (AQ-D) (Gambina et al., 2015). In accordance with literature, the diagnosis of anosognosia was based upon a caregiver-patient discrepancy of at least 2 points in 4 or more items (P: patient; Cg: caregiver).

At T2 visit, in January 2018, relatives reported stability of her memory deficits and a slight reduction of confabulations. MMSE was 30/30 and NPI 12/144 (irritability and apathy predominant sub-items). On this occasion, we performed a lumbar puncture: β42-amyloid 504 pg/ml, p-tau 181 74 pg/ml, and t-tau 340 pg/ml. Cutoffs for abnormal biomarker values routinely used in our laboratory are Aβ42 < 600 pg/ml, t-Tau > 350 pg/ml, and p-Tau181 > 60 pg/ml. In consideration of the patient age, we decided not to perform a cerebral amyloid-PET. A diagnosis of mild amnestic mild cognitive impairment (MCI) due to AD was thus posed (Albert et al., 2011).

At T3 visit, in October 2018, a greater impairment in spatial orientation and a little reduction of autonomies was reported by her husband (she needed assistance for shopping, planning household and administrative activities – iADL6/8, ADL 6/6). Increased apathy and lack of interest in her hobbies were also reported (NPI 14/144 irritability and apathy predominant sub-items), while incidence of spontaneous confabulations was reduced. MMSE was normal (27.86/30, corrected score). On this occasion, we administered neuropsychological tests (Table 1, assessment at follow-up) and the CB to the patient, who produced provoked confabulation in only five questions (Table 2). Interesting also is that A.R. had no awareness of her memory impairment and reduced performance in daily life activities, as confirmed by the AQ-D (Table 1).

**TABLE 2:** A.R. and A.G.’s performances on each of the 11 domains of the Confabulation Battery assessed at T3 visit.

	Correct		Confabulation		Wrong		I don’t know	
	A.R.	A.G.	A.R.	A.G.	A.R.	A.G.	A.R.	A.G.
**Temporal Consciousness**								
Episodic Memory	12	15	3	0	0	0	0	0
Orientation in Time and Place	15	15	0	0	0	0	0	0
Episodic Plans	15	15	0	0	0	0	0	0
**Knowing Consciousness**								
Personal Semantic Memory	15	15	0	0	0	0	0	0
Linguistic Semantic Memory	15	15	0	0	0	0	0	0
Recent General Semantic Memory	15	15	0	0	0	0	0	0
Contemporary General Semantic Memory	13	15	2	0	0	0	0	0
Historical General Semantic Memory	15	15	0	0	0	0	0	0
Semantic Plans	15	15	0	0	0	0	0	0
“I don’t know” Semantic	15	15	0	0	0	0	0	0
“I don’t know” Episodic	15	15	0	0	0	0	0	0

*The CB consists of 165 questions exploring 11 domains belonging to Temporal Consciousness (TC), which refers to awareness of personal past, present, and future, and Knowledge Consciousness (KC), which refers to impersonal knowledge). A.R. produced three confabulations in Episodic Memory Questions (two habit confabulations and one misplacement) and two confabulations in Contemporary General Semantic Memory (one memory confusion and one memory fabrication). For the detailed questions belonging to each domain and scoring instructions, see Dalla Barba et al. (2019).*

### Case 2

A.G.’s first visit was in January 2016 (T1), at the age of 58. He was an office worker with 13 years of education. His medical history was unremarkable, and he took no medications. He has been complaining about memory impairment for some months with difficulties at work. His relatives reported also spontaneous confabulations and reckless behaviors, such as high-speed driving.

At the visit, neurological examination was normal, MMSE was 21.99/30 (corrected score), and NPI was 6/144 (irritability and disinhibition predominant sub-items). During the visit, he reported to physicians that his wife had a lover and administered him sleeping pills every night in order to spend time with her alleged lover.

**FIGURE 2:**
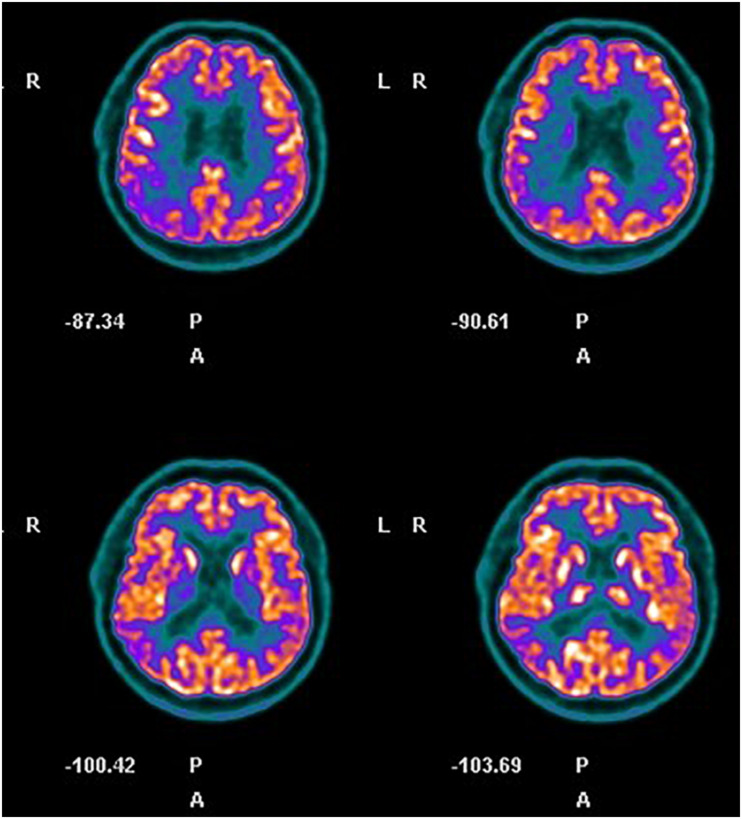
Case 2, cerebral FDG-PET. Hypometabolism in right parietal and temporal areas and at the anterior cingulate cortex is evident.

Neuropsychological assessment highlighted an impairment in executive functions (Table 1, assessment at baseline), raising the suspicion for FTD. Brain MRI showed diffuse mild bilateral and symmetric cortical atrophy; cerebral PET-FDG highlighted wide and moderate hypometabolism in parietal and temporal areas, mainly on the right side and at the anterior cingulate cortex (Figure 2). The amyloid-PET performed subsequently resulted positive. A diagnosis of AD was posed.

In December 2016 (T2), MMSE was 19.99/30 (raw score) and NPI was 5/144 (sub-item irritability and disinhibition). He reported progressive worsening of his memory deficits, which led to job downgrading, but maintaining a strong awareness of his memory impairment as also witnessed by the AQ-D scores (Table 1). Besides, his spontaneous confabulations were reported to be still florid by relatives and friends (i.e., he was certain that his sister was stealing from his house).

In October 2018 (T3), neuropsychological tests comprehensive of the CB were administered (Table 1, assessment at follow-up): he answered correctly all the questions, showing a complete absence of provoked confabulations (Table 2), in contrast with the persistence of spontaneous confabulations. MMSE was 22.49/30 (raw score) and NPI was unchanged. Interesting to observe is that his confabulations probably draw on reality: i.e., his wife filed for divorce shortly after the symptom onset and his sister took regularly care of house working his house, probably moving objects.

## Discussion

According to the NIA-AA criteria for the diagnosis of AD, along with the typical amnestic presentation, atypical variants could be identified with language (primary progressive aphasia), visuospatial (posterior cortical atrophy), or executive dysfunction as initial most prominent cognitive deficits (McKhann et al., 2011). Nevertheless, the wide clinical spectrum of patients with a diagnosis of AD could suggest the existence of other atypical variants. A.R. and A.G. represent two different clinical cases, with respectively late and early onset of AD, both presenting spontaneous confabulations at disease onset.

A.R.’s neuropsychological profile is an amnestic MCI with progression to AD. As far as we know, this is the second report in literature of a patient with amnestic MCI showing spontaneous confabulations (Abbate et al., 2016).

The neuropsychological assessment of A.G. highlighted a predominant impairment in frontal functions posing the matter of a differential diagnosis with FTD. Nevertheless, biomarkers assessment supported a diagnosis of AD (McKhann et al., 2011), and a diagnosis of frontal variant of AD was eventually made. Noteworthy for the differential diagnosis between AD and FTD is that behavioral alterations appear normally later in AD patients in comparison to memory impairment and social behavior is generally appropriate (Kohler et al., 2016). Moreover, patients with FTD show lower awareness of their deficits in comparison to AD patients (DeLozier and Davalos, 2016) and tend to confabulate more (Nedjam et al., 2004). In this respect, A.G. represents a peculiar case with features both of AD, such as the hypometabolism distribution in FDG-PET imaging and the preserved consciousness of his memory impairment, and FTD, such as florid spontaneous confabulations behavioral disturbances at the onset.

The CB allows to detect only provoked confabulations whereas A.G. exhibited mostly spontaneous confabulations for which a validated formal assessment is not available. Previous studies demonstrated no significant correlation between spontaneous and provoked confabulations (Kessels et al., 2008; El Haj and Laroi, 2017) in agreement with the notion that both types of confabulation are dissociated (Kopelman, 1987). However, taking into account that provoked confabulations are more frequent in the early phase of AD, we could not exclude that performing CB earlier than T3 in A.G. could have detected a higher number of provoked confabulations.

Brain circuits involved in spontaneous confabulations are wide, involved in particular posterior orbitofrontal cortices (Turner et al., 2008) and anterior limbic structures (Abbate et al., 2016), including the default mode network (DMN) (Catani et al., 2013). The DMN, diffusely localized bilaterally in the parietal and temporal cortices and in the medial prefrontal cortex (Raichle, 2015), is active during the “resting state” and his activity decreases during the execution of goal-directed tasks (Catani et al., 2013). Among the functions of this system are included consolidation of memory, sampling of external stimuli, and connection between emotional and cognitive processes (Mohan et al., 2016). A decreased connectivity between the posterior (precuneus and posterior cingulate cortex) and anterior (anterior cingulate cortex and medial prefrontal cortex) regions of the DMN has been already associated with AD (Mohan et al., 2016). According to recent evidences, the insufficient suppression of the DMN during cognitive tasks is responsible for the inability to distinguish accurate information from the irrelevant ones during the process of memory retrieval, thus contributing to generate confabulations (Venneri et al., 2017).

There are currently two major theories about the neurocognitive mechanisms beyond confabulations. According to the Memory, Consciousness and Temporality Theory (Dalla Barba, 2001), confabulations reflect an impairment of the Temporal Consciousness (TC) rather than the Knowing Consciousness (KC). In this model, the hippocampus acts as a pointer to create a personal temporal framework of information. When the hippocampus unilaterally receives distorted information from extra-hippocampal areas, confabulations emerge, while bilateral hippocampal damage is linked to amnesia without confabulations (Dalla Barba et al., 2020). From this perspective, the major disruption of personal temporality in FTD in comparison to AD may be responsible for the higher frequency of confabulations (Nedjam et al., 2004; Bajo et al., 2017). Besides, according to this theory, episodic memory should be the most affected domain of the CB, since it involves autobiographical memory, and habit confabulations should be the most frequent.

On the other hand, according to the Strategic Retrieval Hypothesis, confabulations can be considered more a deficit in retrieval rather than in encoding processes, being related to damage of the ventromedial prefrontal cortex and involving particularly semantic memory (Gilboa et al., 2006).

As seen in Table 2, provoked confabulations were evident only in A.R. and could be classified as habits in only two cases out of five. Besides, episodic memory and semantic memory (and so TC and KC sections) were equally involved. These results seem to suggest that confabulations are not directly related to damage of a single brain area, but rather have a complex neuroanatomical origin. This is in line with recent studies which have linked the confabulation tendency in AD patients to the impairment of complex circuits between different hubs, particularly between the right prefrontal cortex and the mediotemporal regions involved in memory retrieval (Venneri et al., 2017).

Cerebral PET-FDG images highlighted both the absence of a clear detriment of frontal regions and a slight prevalence of the hypometabolism on the right side in both patients.

While the left hemisphere is responsible for production and comprehension of language, the right hemisphere plays an equally important role in language function, being in charge of prosodic and paralinguistic aspects of speech, interpretation of emotional contents, and novelty detection. In this respect, hypofunction of the right hemisphere has been linked to delusions and confabulations (Gurin and Blum, 2017). The defective function of the right hemisphere *per se* does not originate false thoughts, but rather the reduced inhibitory control of the right lobes over the left hemisphere allows confabulatory explanations to emerge. Inside the right hemisphere, the two areas involved in this strict control of the contralateral hemisphere are the temporal and frontal lobes. A degenerative process that impairs the connections between these two hubs allows the left hemisphere to emerge, leading to misinterpretation of reality and thus to confabulatory tendency.

Interesting to notice is that A.G. produced a kind of false claims posing the matter of the differential diagnosis between confabulations and delusions, both resulting from the impairment of a common set of processes involved in monitoring and evaluation of thoughts (Turner and Coltheart, 2010). The nature of false thoughts leads us toward a diagnosis of confabulations rather than delusions: A.G. did not show neither jealousy toward his wife in other situations of daily life nor rage against the sister, but in both cases he was just worried. Besides, his thoughts seemed to have some basis in reality. For these reasons, the diagnosis of delusion seemed to be less probable.

According to the two-factor theory, false thoughts require a combination of two factors to emerge where the first one is a neuropsychological impairment that prompts the false belief and the second one involves a deterioration of the checking system. In this view, confabulations and delusions share an impairment in the unconscious checking system: in the absence of a system that tags novel thoughts that require an additional conscious checking, ideas deriving from imagination are perceived as true (Coltheart, 2010). What differs between confabulations and delusions is the first factor; that is, confabulations require an impairment in memory retrieval. However, it should be also considered that certain forms of delusions could evolve from confabulations (Kopelman, 2010). Thus, delusions and confabulations could be considered as two sides of the same coin considering their common neuropsychological basis and could not always be distinguished.

Finally, we analyzed the relationship between confabulatory phenomena and self-awareness. The anatomical underpinning of self-consciousness has long thought to be the frontal lobe specifically on the right side (Miller et al., 2001). Nevertheless, it is nowadays meant to involve a larger network including fronto-orbital cortex, precuneus/posterior cingulate gyrus, temporo-parietal junctions, and temporal poles (Arroyo-Anllo et al., 2016). Self-consciousness and reality evaluation processes are thus regulated by common anatomical areas, involving in particular the DMN and the right hemisphere. In this view, it has been hypothesized that the lack of insight could act as facilitator for confabulations and delusional thoughts (Harwood et al., 2005). Generally, FTD patients show an earlier reduction of insight than AD ones (Arroyo-Anllo et al., 2016). On the other hand, anosognosia in amnestic MCI patients is associated with higher amyloid burden and to a higher risk of conversion to AD within 2 years (Therriault et al., 2018).

In this perspective, our patients represent two atypical cases in which this correspondence between disease awareness and confabulations is not respected. A.R. had no consciousness of her memory deficits since disease onset; as time passes, she maintained her lack of awareness while confabulations progressively reduced. On the contrary, A.G. had a clear consciousness of his memory impairment since disease onset despite having a clinical and neuropsychological profile resembling behavioral variant of FTD. He had also florid confabulations which persisted and even increased over time. The absence of correspondence between confabulation and disease awareness could be explained by behavioral aspects. Indeed, recent models aimed to explain processes underlying self-awareness in neurodegenerative disease put out the role of motivational and emotional factor (Rosen, 2011). Several previous studies found a relationship between anosognosia and apathy in AD and in MCI (Rosen, 2011; Shany-Ur et al., 2014; Jacus, 2017).

We must take into consideration the limitations of this report, among them the small number of patients and the lack of a longitudinal confabulation assessment through CB, which was administered only once. Our results thus need further confirmation by other studies aimed at detected features of confabulations in AD patients.

## Conclusion

In conclusion, we hypothesize that spontaneous confabulations can be considered a rare but possible clinical presentation of typical AD or an atypical variant of AD; they could also contribute to identify converter MCI patients to AD at early stages of the disease.

Besides, our results seem to support the complex neuropathology beyond confabulations, which can rarely be attributed to the damage of a single brain area, rather being the result of a complex interaction between crucial hubs.

We also speculate that the neural circuits behind confabulations and self-awareness share many components, mainly localized in the right hemisphere, but this is not sufficient to consider them as codependent and necessarily associated dimensions.

## Data Availability Statement

All datasets presented in this study are included in the article/supplementary material.

## Ethics Statement

Written informed consent was obtained from the individual(s) for the publication of any potentially identifiable images or data included in this article.

## Author Contributions

EB, VN, and GT contributed to the conception and design of the study. EB wrote the first draft of the manuscript. VN, GT, and RC wrote sections of the manuscript. CR, JB, and SC performed the neuropsychological assessments. All authors contributed to manuscript revision, read, and approved the submitted version.

## Conflict of Interest

The authors declare that the research was conducted in the absence of any commercial or financial relationships that could be construed as a potential conflict of interest.
